# A comparative study of incidence rate and severity of influenza virus and respiratory syncytial virus associated hospitalisation in older adults in Jiangsu Province, China: a retrospective analysis of a regional medical database

**DOI:** 10.7189/jogh.16.04115

**Published:** 2026-03-27

**Authors:** Tiantian Zhang, Ling Guo, Yumeng Miao, Caini Wang, Xin Wang, You Li

**Affiliations:** 1 Department of Epidemiology, National Vaccine Innovation Platform, School of Public Health, Nanjing Medical University, Nanjing, China; 2 Department of Epidemiology, School of Public Health, Key Laboratory of Public Health Safety and Emergency Prevention and Control Technology of Higher Education Institutions in Jiangsu Province, Nanjing Medical University, Nanjing, China; 3 Kunshan Women and Children's Health Care Hospital, Suzhou, China; 4 Department of Biostatistics, National Vaccine Innovation Platform, School of Public Health, Nanjing Medical University, Nanjing, China; 5 Changzhou Third People's Hospital, Changzhou Medical Centre, Nanjing Medical University, Changzhou, China; 6 Centre for Global Health, Usher Institute, University of Edinburgh, Edinburgh, UK

## Abstract

**Background:**

Few studies have compared the disease burden between influenza virus (IFV) and respiratory syncytial virus (RSV) in older adults, particularly outside high-income countries. We aimed to compare IFV- and RSV-associated hospitalisation burden and severity in older adults in Jiangsu Province, China.

**Methods:**

Drawing on a medical database from Jiangsu Province, an economically-developed province in eastern China, we included acute respiratory infections (ARI) admission records in adults aged ≥60 years during 2020 to 2023, linked with viral testing data, where available. We applied a time-series regression model for estimating RSV- and IFV-associated hospitalisation rates by year, age group, and sex. We compared disease severity, defined as a composite of mechanical ventilation use, intensive care unit (ICU) admission, or in-hospital mortality, between RSV- and IFV-positive cases using multivariable logistic regression, with adjustments for age, sex, year, region, and laboratory method.

**Results:**

We included 188 529 ARI hospitalisation episodes during the study period. RSV and IFV had comparable hospitalisation rates, peaking in 2023 at 98 per 100 000 person-years (95% confidence interval (CI) = 81–115) for RSV and 116 per 100 000 person-years (95 CI = 100–133) for IFV. Hospitalisation rates increased with older age and were marginally higher in males. The risk for severe cases was 1.47 (95% CI = 1.086–1.997, *P* = 0.013) times higher in RSV than in IFV. Older age and being males were independent risk factors for severe cases.

**Conclusions:**

We found the RSV hospitalisation burden to be comparable to that of IFV in older adults, albeit with higher severity. The findings contribute to the evidence base for recommendation of RSV and IFV vaccination in older adults in China.

Influenza virus (IFV) and respiratory syncytial virus (RSV) are important causes of acute respiratory infections (ARI) in older adults [1]. As both diseases are vaccine-preventable among older adults [2,3], there is a need for research comparing the disease burden of IFV and RSV in older adults to inform vaccine recommendations. However, existing studies among older adults mostly focused on IFV or RSV individually, while heterogeneities across study population, time period, setting, and diagnostic and analytical approaches hinder comparison. Moreover, few studies have reported on the disease burden of RSV in older adults in low- and middle-income countries, specifically.

Although IFV vaccines are available in China via the private market, some regions recently started to offer it freely older adults. For example, Taizhou (Jiangsu Province) launched such a programme in the 2023–24 winter season targeting older adults aged ≥65 years [4]. Simultaneously, a phase-III clinical trial of RSV vaccine for older adults (NCT06551181) was ongoing in China [5,6], with licensure expected in next 2–3 years. However, to our knowledge, no studies have reported on the incidence rate of RSV diseases among older adults in China.

In this study, we utilised a regionally representative medical database from China covering over 200 hospitals in Jiangsu Province, and linked diagnostic information with laboratory test results. We compared the hospitalisation burden and disease severity of IFV and RSV within the same population over the same period. Our aim was to fill the evidence gap on the RSV disease burden in older adults in China to help inform vaccine strategies in the future.

## METHODS

### Study design

This study adopted two epidemiologic designs in this study. First, to estimate the hospitalisation burden associated with IFV and RSV, we used an ecological study design which allowed us to assess the association of weekly time series of aggregated IFV and RSV cases with non-COVID-19 ARI hospitalisation cases at the population level. For disease severity of IFV and RSV, we employed a logistic regression approach to compare the likelihood of severe illness between hospitalised IFV and RSV cases. The study period spanned four years (2020–2023); non-pharmaceutical interventions (NPIs) were in place for most of the time during 2020 to 2022, and had been lifted since December 2022.

### Data source

We used a regional medical database which included routine medical data from more than 200 hospitals in Jiangsu Province, covering more than 95% of grade III hospitals province-wide (the most common grade of hospitals with beds) from 96 counties in all 13 municipalities. More details on the data governance, storage, and processing within this database can be found elsewhere [7].

Here, we extracted de-identified individual patient-level hospital admission records due to non-COVID-19 ARI spanning the period from 1 January 2020 to 31 December 2023 based on date of admission. We identified non-COVID-19 ARI cases based on the following related ICD 10 codes in the primary diagnosis (Table S1 in the **Online Supplementary Document**): acute upper respiratory tract infections (J00–J06), influenza and pneumonia (J09–J18), and other acute lower respiratory tract infections (J20–J22). We further restricted the admission records to patients aged ≥60 years who resided in Jiangsu Province. For each patient, we excluded re-admissions that occurred within the first 14 days following the previous admission, as they were likely due to the same ARI episode [8,9]; we replaced the criteria of 14 days with 7 days and 30 days as sensitivity analysis. We set no restrictions to the admission source (*e.g.* first admission, inter-hospital transfer).

For these eligible patients, we extracted all laboratory testing records for respiratory viruses if the test was conducted within one week (before or after) for the same individuals (regardless of whether the test was conducted in the same hospital). In Jiangsu Province, testing for respiratory viruses was ordered at the discretion of clinicians and the diagnostic tests varied substantially by individual hospitals. We excluded serology tests of IgG, as they could not be used to confirm recent infections. Moreover, considering the variations in testing practices, in cases where there were two or more laboratory testing approaches for the same virus for the same episode of admission, we prioritised the inclusion of polymerase chain reaction (PCR) testing data over other non-PCR testing approaches, selecting only the earliest test if multiple tests of the same category (PCR *vs*. non-PCR) were available. We categorised cases where we could not determine whether a PCR test was conducted or not based on available information (although we could rule out serology) as missing data. We analysed IFV A and B subtypes together as IFV, and counted co-infections (RSV and IFV both positive within the same episode) in the respective totals for each virus.

Besides medical data, we extracted the population data from Jiangsu Provincial Statistical Yearbook [10] and the National Bureau of Statistics [11] to estimate the hospitalisation rates. We converted all dates to ISO weeks to ensure consistency across data sets.

### Statistical analysis

#### Hospitalisation rate

Based on the above-described data on ARI admission and laboratory testing, we generated the weekly time series of aggregated number of ARI and those with laboratory-confirmed infections by IFV, RSV, and parainfluenza virus (for use in sensitivity analysis) separately. Following previous research [12,13], we constructed a time-series linear regression model to assess the number of ARI hospitalisations that could be potentially explained by IFV and RSV using the following formula:

*ARI_t_* = β_0_ + β_1_*RSV_t_* + β_2_*IFV_t_* + *ϵ_t_*

Here, *ARI_t_* indicates the number of ARI hospitalisations at time t, *β_0_* the constant term, *RSV_t_* the number of RSV positives at time *t*, *IFV_t_* the number of IFV positives at time *t*, and *ϵ_t_* the error term. This allowed us to predict the number of ARI hospitalisations for each week by setting *RSV_t_* to what was observed in that week, and the number of ARI hospitalisations for each week by setting *RSV_t_* to zero. Then, the difference between the two predicted numbers for each week was the number of hospitalisations associated with RSV. These weekly numbers of RSV-associated hospitalisation were subsequently summed up for obtaining the annual number and incidence rate of RSV-associated hospitalisation for the year of 2020, 2021, 2022 and 2023, separately. We used the same approach to estimate IFV-associated hospitalisation number and incidence rate.

Following the relaxation of NPIs, there was a major COVID-19 outbreak during week 50, 2022, to week 3, 2023, in Jiangsu Province, which hindered the differentiation of COVID-19 from non-COVID-19 ARI cases in all hospitals. Therefore, we removed this six week period from model fitting although this did not affect the inclusion of data on IFV and RSV positives for predicting the number of ARI hospitalisations during this period (we completely removed this period for model prediction as a sensitivity analysis).

By applying the above method, we estimated RSV-associated and IFV-associated ARI hospitalisation rate for the three predefined age groups, separately (60–69, 70–79, and ≥80 years). Subsequently, we estimated RSV-associated and IFV-associated ARI hospitalisation rates for the overall age group of ≥60 years by totalling up the number of hospitalisations across the three age groups (rather than by fitting a separate model). As the model assumed that the relationship between viral positives and ARI admissions was linear and separable for RSV and IFV, and that there was no confounding by other co-circulating respiratory viruses or secular drivers, the estimates derived here should be interpreted as the fraction of ARI hospitalisations statistically associated with each virus under the model assumptions.

We further conducted subgroup analysis by sex, followed by one-way sensitivity analyses to evaluate the robustness of the model (**Online Supplementary Document**). In the first sensitivity analysis, we added a natural cubic spline function to index of week for controlling time-related trends. In the second sensitivity analysis, we added the weekly number of parainfluenza virus cases as a model covariate. In the third analysis, we further restricted the included ARI cases to acute lower respiratory infection (ALRI). In the last sensitivity analysis, we removed the period of a major COVID epidemic during week 50, 2022 to week 3, 2023 from model prediction.

#### Disease severity

We selected hospitalised ARI cases with positive testing results for IFV or RSV to compare their disease severity. We defined severe disease as any duration of mechanical ventilation or ICU admission, or any recorded in-hospital mortality. We did not use in-hospital mortality as a single severity indicator, since families of patients who were about to die tended to request to send patients home and those deaths were not recorded in the hospital database, resulting in underestimation. We used logistic regression to assess the association between virus type and severe disease, adjusting for age group, sex, year of hospitalisation, geographic region within Jiangsu province, and laboratory method.

All analyses were performed in *R*, version 4.4.1 (R Foundation for Statistical Computing, Vienna, Asutria).

## RESULTS

**Figure 1:**
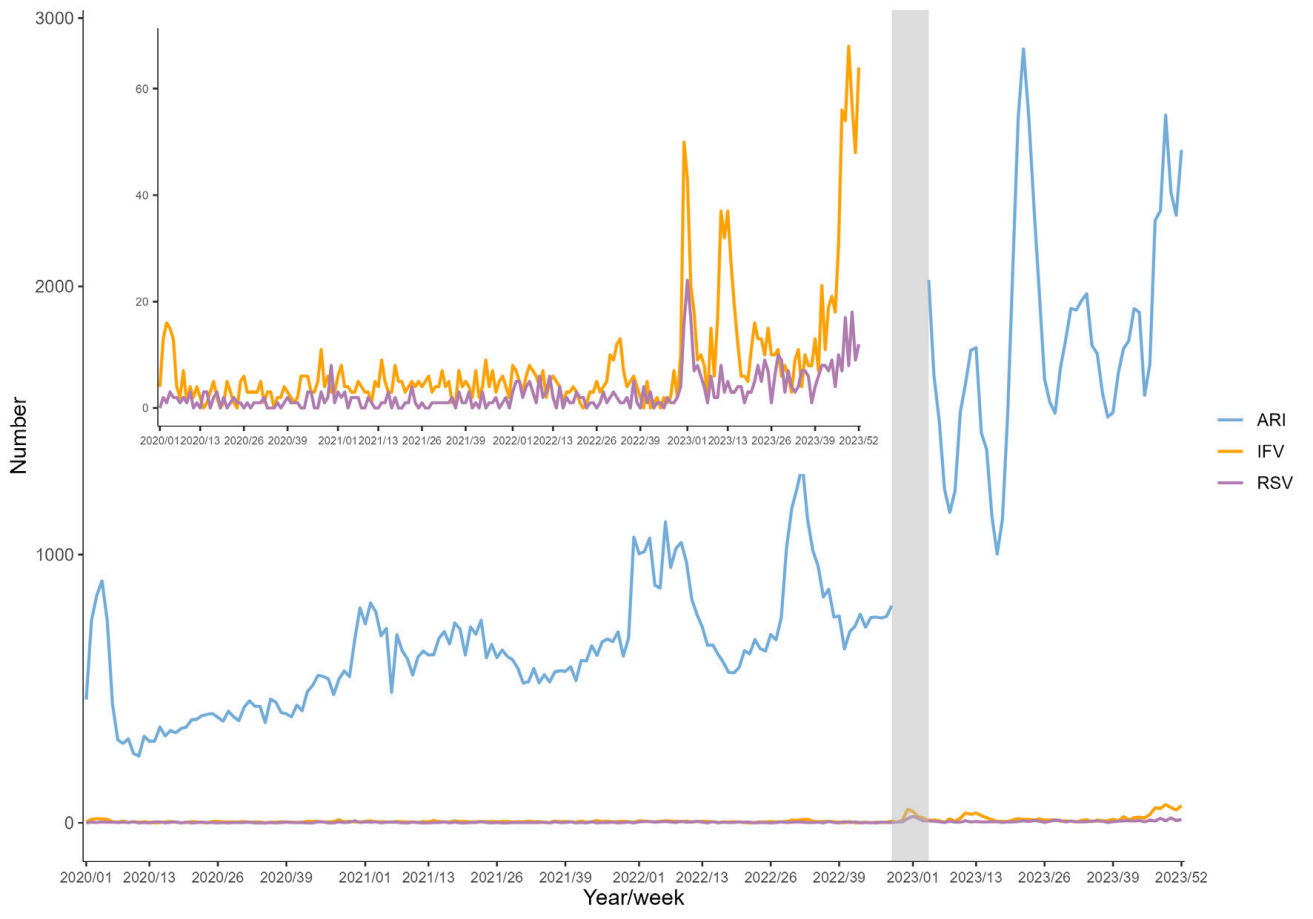
Weekly aggregated number of non-COVID-19 ARI hospitalisations, RSV positives, and IFV positives in the study population. Shaded area denoted the local major COVID-19 pandemic period when the data on ARI hospitalisations were excluded, as COVID-19 cases could not be ruled out. ARI – acute respiratory infections, COVID-19 – coronavirus disease 2019, IFV – influenza virus, RSV – respiratory syncytial virus.

We identified 188 529 episodes of ARI hospitalisation from January 2020 to December 2023, of which 594 were positive for RSV and 1726 for IFV. Both the number of ARI hospitalisation and the number of RSV and IFV positives increased substantially following the relaxation of NPIs in late 2022 (**Figure 1**; Table S2 in the **Online Supplementary Document**). There were more males than females among RSV and IFV cases, while RSV positive cases were mostly older than IFV positive cases. A larger proportion of IFV cases were confirmed by PCR than RSV. There were also geographical variations by region, with northern Jiangsu having the highest proportion of RSV cases (74.2%) and southern Jiangsu having the highest proportion of IFV cases (45.8%) (**Table 1**).

**Table 1:** Basic characteristics of RSV and IFV positive cases in the study

	RSV positive (n = 594)	IFV positive (n = 1726)
**Sex**		
Male	340 (57.2)	978 (56.7)
Female	251 (42.3)	748 (43.3)
Missing	3 (0.5)	0 (0)
**Age in years, mean (standard deviation)**	76.1 (9.0)	74.7 (9.4)
**Age group in years**		
60–69	158 (26.6)	600 (34.8)
70–79	223 (37.5)	567 (32.9)
≥80	213 (35.9)	559 (32.4)
**Time period**		
January 2020 to December 2020	69 (11.6)	214 (12.4)
January 2021 to December 2021	60 (10.1)	232 (13.4)
January 2022 to December 2022	121 (20.4)	266 (15.4)
January 2023 to December 2023	344 (57.9)	1014 (58.7)
**Laboratory test**		
PCR	47 (7.9)	368 (21.3)
Non-PCR	374 (63)	909 (52.7)
Missing	173 (29.1)	449 (26)
**ICU admission**		
Yes	82 (13.8)	139 (8.1)
No	512 (86.2)	1587 (91.9)
**Ventilation use**		
Yes	20 (3.4)	41 (2.4)
No	574 (96.6)	1685 (97.6)
**Death in hospital**		
Yes	9 (1.5)	37 (2.1)
No	585 (98.5)	1689 (97.9)
Region*		
Northern Jiangsu	441 (74.2)	570 (33)
Central Jiangsu	70 (11.8)	293 (17)
Southern Jiangsu	68 (11.4)	790 (45.8)
Missing	15 (2.5)	73 (4.2)

ALRI – acute lower respiratory infections, AURI – acute upper respiratory infections, IFV – influenza virus, PCR – polymerase chain reaction, RSV – respiratory syncytial virus

*Northern Jiangsu consists of Xuzhou, Lianyungang, Suqian, Huai’an, and Yancheng. Middle Jiangsu consists of Yangzhou, Taizhou and Nantong. Southern Jiangsu consists of Nanjing, Zhenjiang, Changzhou, Wuxi, and Suzhou.

### Hospitalisation rate

The age-specific time-series models demonstrated good model fits, with adjusted *R*^2^ of 0.55, 0.48, and 0.47 in the models of 60–69 years, 70–79 years, and ≥80 years, respectively.

When NPIs were in place between 2020 and 2022, hospitalisation rates among adults aged ≥60 years were relatively low for both RSV and IFV, ranging from 18 to 35 per 100 000 person-years for the former and from 27 to 31 per 100 000 person-years for the latter. When all NPIs were lifted in 2023, hospitalisation rates (per 100 000 person-years) increased significantly for both RSV (98; 95% CI = 81–115) and for IFV (116; 95% CI = 100–133). Both RSV- and IFV-associated hospitalisation rates increased with increased age in each year (**Table 2**).

**Table 2:** RSV- or IFV-associated ARI hospitalisation rates in older adults in Jiangsu, stratified by time periods

	Hospitalisation rate (95% CI) per 100 000 person-year	
**Age groups in years**	**RSV**	**IFV**
January 2020 to December 2020		
*60–69*	11 (7–14)	17 (14–20)
*70–79*	30 (21–38)	32 (23–41)
*≥80*	43 (27–58)	50 (36–63)
*≥60*	21 (18–25)	27 (23–31)
January 2021 to December 2021		
*60–69*	9 (6–11)	22 (18–26)
*70–79*	27 (19–34)	29 (21–37)
*≥80*	30 (19–41)	48 (34–60)
*≥60*	18 (15–21)	28 (24–32)
January 2022 to December 2022		
*60–69*	15 (11–19)	22 (18–26)
*70–79*	51 (36–64)	31 (22–38)
*≥80*	68 (43–91)	66 (48–83)
*≥60*	35 (28–41)	31 (27–36)
January 2023 to December 2023		
*60–69*	48 (34–62)	77 (62–91)
*70–79*	123 (88–155)	117 (84–147)
*≥80*	207 (131–278)	248 (178–311)
*≥60*	98 (81–115)	116 (100–133)

ARI – acute respiratory infections, CI – confidence interval, IFV – influenza virus, RSV – respiratory syncytial virus

**Figure 2:**
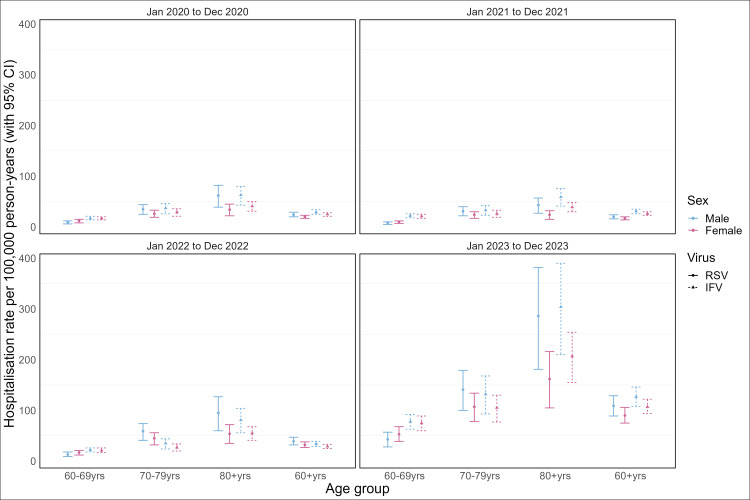
RSV or IFV-associated ARI hospitalisation rates by sex and age among older adults. ARI – acute respiratory infections, IFV – influenza virus, RSV – respiratory syncytial virus, yrs – years.

Hospitalisation rates were generally similar between IFV and RSV, despite a slightly higher point estimate for IFV in most years. The only exception was in the 60–70-year-old age group, where a statistically higher IFV-associated hospitalisation rate was observed in 2020, 2021, and 2023 (**Table 2**). Further subgroup analysis by sex did not yield statistically significant differences in the hospitalisation rates of IFV or RSV between males and females, despite a higher, yet statistically non-significant hospitalisation rates in males than females for the ≥70-year-old age group (**Figure 2**; Table S3 in the **Online Supplementary Document**).

We arrived at broadly similar estimates in sensitivity analysis, with the only exception emerging from the sensitivity analysis that applied a natural cubic spline to the index of weeks, which yielded lower estimates across age groups and time period for both RSV and IFV (Figure S1 in the **Online Supplementary Document**).

### Disease severity

RSV infection remained associated with a significantly higher risk of severe disease compared with IFV infection (odds ratio (OR) = 1.47; 95% CI = 1.08–2.00). The odds of severe disease increased significantly with age, particularly among ≥80-year-olds (OR = 1.76; 95% CI = 1.24–2.48). Female sex was associated with lower odds of severe disease (OR = 0.58; 95% CI = 0.43–0.77). Hospitalisation in 2021 and 2023 was associated with significantly lower severity compared with 2020, while residing in Central Jiangsu was also associated with reduced severity risk. Laboratory method was not significantly associated with disease severity in the final model. While the proportion of severe cases was higher for RSV than for IFV in central Jiangsu, the two viruses had comparable severity proportions in northern and southern Jiangsu (**Table 3**).

**Table 3:** Factors associated with severe cases among RSV- or IFV-positive hospitalised older adults in Jiangsu by multivariate logistic regression analysis*

	OR (95% CI)	*P*-value
**Virus type**		
*IF*		
*RSV*	1.472 (1.086–1.997)	0.013
**Age group in years**		
*60–69*		
*70–79*	1.219 (0.852–1.743)	0.281
*≥80*	1.756 (1.242–2.483)	0.001
**Sex**		
*Male*		
*Female*	0.576 (0.431–0.769)	<0.001
**Time period**		
January 2020 to December 2020		
January 2021 to December 2021	0.576 (0.336–0.987)	0.045
January 2022 to December 2022	1.143 (0.728–1.795)	0.560
January 2023 to December 2023	0.573 (0.381–0.862)	0.007
**Region†**		
*Northern Jiangsu*		
*Central Jiangsu*	0.474 (0.298–0.754)	0.002
*Southern Jiangsu*	0.944 (0.648–1.374)	0.764
**Laboratory test**		
*Missing*		
*Non-PCR*	0.823 (0.594–1.139)	0.240
*PCR*	1.328 (0.853–2.068)	0.210

CI – confidence interval, IFV – influenza virus, OR – odds ratio, PCR – polymerase chain reaction, RSV – respiratory syncytial virus

*Severe cases were defined as the use of mechanical ventilation, admission to intensive care unit, or death.

†Northern Jiangsu consists of Xuzhou, Lianyungang, Suqian, Huai’an, and Yancheng. Middle Jiangsu consists of Yangzhou, Taizhou and Nantong. Southern Jiangsu consists of Nanjing, Zhenjiang, Changzhou, Wuxi, and Suzhou.

## DISCUSSION

To the best of our knowledge, this is the first study to estimate the RSV-associated hospitalisation burden in older adults in China and provide a comparison of the hospitalisation burden between RSV and IFV. Our findings show comparable hospitalisation rates between RSV and IFV among the study population overall. The proportion of severe cases, as indicated by use of mechanical ventilation, ICU admission, and death, was approximately 1.47 times higher in RSV than IFV, particularly in adults aged ≥80 years. These estimates provide an important evidence base for recommendations in vaccination programmes for RSV and IFV in older adults.

As expected, hospitalisation rates for both RSV and IFV were significantly lower in 2020–2022, which could possibly be associated with the impact of the COVID-19 pandemic and its associated NPIs, compared to the year of 2023 when all NPIs were lifted. The estimated IFV-associated hospitalisation rate in adults aged ≥60 years in this study (116 per 100 000 person-years; 95% CI = 100–133) was similar to previously published estimates in Beijing before the COVID-19 pandemic, ranging from 92 to 150 per 100 000 person-years [14,15]. This suggests that the hospitalisation burden of IFV had returned to the pre-pandemic level by 2023. The estimated IFV-associated hospitalisation rate in the study population (*i.e.*≥60 years) in 2023 was also broadly comparable to the pre-pandemic estimates for individuals aged ≥65 years from other studies in China (ranging from 95 to 240 per 100 000 person-years) [16–18]. This lends credence to the robustness of our estimates. However, as 2023 represents the first year following the relaxation of NPIs, both the immunological landscape and viral transmission dynamics may still be shifting; surveillance data from later years will be required to establish a stable post-pandemic baseline.

We observed that RSV had generally comparable hospitalisation rates to IFV across the study period, highlighting its substantial disease burden that warrants public health actions in China. Furthermore, we noted that the proportion of severe diseases was approximately 1.47 times higher among RSV cases than IFV cases although this finding varied greatly by region, with comparable proportions of severe cases between RSV and IFV also being observed. While the overall higher proportion of RSV severe cases than IFV in our study was generally consistent with those obtained in research in the USA [19], France [20], and Germany [21], a multi-centre study from Spain [22] suggested that RSV presented similar or lower intrinsic severity, with comparable risks for ICU admission but lower risk for mortality. Despite these mixed results, our findings, together with existing studies, suggest that the overall severity of RSV cases is comparable to that of IFV ones in the older adult population.

We acknowledge the following limitations in this study. First, respiratory viral testing was consistently low during the study period, with variations in testing approaches by individual hospitals. Therefore, we applied an ecological study design for estimating RSV- and IFV-associated hospitalisation rates, rather than relying on the relatively sparse individual-level data with available viral testing results. The low level of respiratory viral testing indicated a relatively limited representativeness for assessing disease severity; we could not fully rule out that disease severity of cases positive for RSV or IFV differed from those who were infected with RSV or IFV, but were not tested. Furthermore, due to the relatively limited data on viral testing, we were unable to construct separate models for assessing regional differences (including urban-rural difference) in the hospitalisation rates. Regional variations in the proportion of severe cases should be interpreted cautiously, as they may reflect differences in testing practices or admission criteria across regions rather than true etiologic differences. Second, we were unable to specify the estimates by IFV vaccination status. Nonetheless, IFV vaccination rate was consistently low over the study period, with estimates ranging from 0.36% to 1.59% for the 2014–2021 period [23] and <1% in the season of 2021–22 [24]. Therefore, our estimates could generally represent the baseline disease burden of IFV in an unvaccinated population. We also acknowledge that all composite severity indicators were subject to variability due to incomplete mortality recording and differences in criteria for ICU admission and initiation of mechanical ventilation over time and across hospitals, particularly during and after the COVID-19 pandemic. This variability may have differentially influenced the comparison of severity between RSV and influenza cases. Third, in-hospital mortality rates were likely underestimated due to discharge against medical advice. An earlier nationwide ecological study [25] in China, for example, found IFV to be associated with approximately 71 000 deaths (in- or out-of-hospital) in adults aged ≥60 years nationwide, equivalent to 38.5 per 100 000 person-years. Finally, we could not estimate hospitalisation rates by comorbidity status, as we did not have the corresponding population denominator data for the calculation (*i.e.* the population with specific comorbidities) or the full records, as we only had access to the primary and secondary diagnosis codes that might not have included all records of comorbidities.

## CONCLUSIONS

By leveraging a regionally well representative medical database in China, we addressed data gaps in the comparison of RSV and IFV-associated hospitalisation burden in China. As adult RSV vaccines are at late clinical development stage in China, with licensure expected in the next 2–3 years, our study provides important evidence on disease burden for health economic evaluation of RSV vaccines in China. We warn, however, that formulating specific vaccination policies still requires integrated decision-making based on multidimensional evidence including vaccine efficacy, cost-effectiveness, and accessibility.

## Additional material


Online Supplementary Document
