# Supplementary material for: A comparative study of incidence rate and severity of influenza virus and respiratory syncytial virus associated hospitalisation in older adults in Jiangsu Province, China: a retrospective analysis of a regional medical database
Source: J Glob Health. 2026 Mar 27;16:04115. doi: 10.7189/jogh.16.04115 (PMC13021056; doi:10.7189/jogh.16.04115)
Supplement: Online Supplementary Document [file jogh-16-04115-s001.pdf]

Supplement to: Zhang T, Guo L, Miao Y, Wang C, Wang X, Li Y. A comparative study of incidence rate and severity of influenza virus and respiratory syncytial virus associated hospitalisation in older adults in Jiangsu Province, China: a retrospective analysis of a regional medical database. J Glob Health. 2026;16:04115.

Contents

Supplementary methods 1
- ICD-10 codes for identifying ARI 1
- Regression formula used in subgroup and sensitivity analyses 1
  - Subgroup analysis by sex 1
  - Sensitivity analysis that used natural cubic spline function 1
  - Sensitivity analysis that added parainfluenza (PIV) as a covariate 1
  - Sensitivity analysis that restricted to ALRI cases 1

Supplementary results 3
- Additional results not reported in the main text 3

List of supplementary tables

Table S1. List of ICD-10 codes for identifying ARI 1

Table S2. Proportion of ARI hospitalisations tested for RSV and IFV by year 3

Table S3. RSV or IFV-associated ARI hospitalisation rates in older adults by sex in Jiangsu 4

List of supplementary figures

Figure S1. Comparison of estimated hospitalisation rates between main and sensitivity analyses 5

Supplementary methods

ICD-10 codes for identifying ARI

Table S1. List of ICD-10 codes for identifying ARI

Disease Condition	ICD-10 codes*
Acute upper respiratory tract infections	J00-J06
Influenza and Pneumonia	J09-J18
Other acute lower respiratory tract infections	J20-J22

ARI = Acute respiratory infections. ICD = International Classification of Diseases. *ICD-10 codes include all the subcodes under the category, e.g., J12 includes all the subcodes of J12.1, J12.9, etc.

Regression formula used in subgroup and sensitivity analyses

Subgroup analysis by sex

$$ARI_t^{sex} = \beta_0^{sex} + \beta_1^{sex} \cdot RSV_t + \beta_2^{sex} \cdot IFV_t + \epsilon_t^{sex} \qquad (S1)$$

ARI_t^{sex}: number of sex-specific ARI hospitalisations at time t. β_0^{sex}: constant term. RSV_t: number of RSV positives at time t. IFV_t: number of IFV positives at time t. ϵ_t^{sex}: error term.

Sensitivity analysis that used natural cubic spline function

$$ARI_t = \beta_0 + \beta_1 \cdot RSV_t + \beta_2 \cdot IFV_t + \sum_{k=1}^{K} \gamma k \cdot Bk(week) + \epsilon_t \qquad (SA1)$$

ARI_t: number of ARI hospitalisations at time t. β_0: constant term. RSV_t: number of RSV positives at time t. IFV_t: number of IFV positives at time t. Bk(week): natural spline basis function. γk: coefficients of the basis function. ϵ_t: error term.

Sensitivity analysis that added parainfluenza (PIV) as a covariate

$$ARI_t = \beta_0 + \beta_1 \cdot RSV_t + \beta_2 \cdot IFV_t + \beta_3 \cdot PIV_t + \epsilon_t \qquad (SA2)$$

ARI_t: number of ARI hospitalisations at time t. β_0: constant term. RSV_t: number of RSV positives at time t. IFV_t: number of IFV positives at time t. PIV_t: number of PIV positives at time t. ϵ_t: error term.

Sensitivity analysis that restricted to ALRI cases

$$ALRI_t = \beta_0 + \beta_1 \cdot RSV_t^{ALRI} + \beta_2 \cdot IFV_t^{ALRI} + \epsilon_t \qquad (SA3)$$

$ALRI_t$: number of ALRI hospitalisations at time t. β_0: constant term. RSV_t^{ALRI}: number of RSV positives among ALRI at time t. IFV_t^{ALRI}: number of IFV positives among ALRI at time t. ϵ_t: error term.

Supplementary results

Additional results not reported in the main text

Table S2. Proportion of ARI hospitalisations tested for RSV and IFV by year

Year	ARI (n)	ARI tested for RSV (n, %)	ARI tested for IFV (n, %)
2020	23879	3805, 14.4%	3432, 15.9%
2021	33604	4445, 13.2%	3898, 11.6%
2022	40540	8283, 17.6%	7141, 20.4%
2023	88495	15401, 17.4%	17324, 19.6%

RSV = respiratory syncytial virus. IFV = influenza virus. ARI = acute respiratory infections.

Table S3. RSV or IFV-associated ARI hospitalisation rates in older adults by sex in Jiangsu

Year	Age group	Sex	Hospitalization rate (95%CI), per 100,000	
			**RSV**	**IFV**
Jan 2020 to Dec 2020	60-<70 years	Male	9 (6, 12)	17 (14, 21)
		Female	12 (8, 15)	17 (14, 20)
	70-<80 years	Male	35 (25, 44)	37 (26, 46)
		Female	26 (19, 33)	29 (21, 36)
	≥80 years	Male	62 (39, 82)	63 (43, 80)
		Female	34 (22, 45)	41 (31, 50)
	**≥60 years**	**Male**	**24 (20, 29)**	**29 (25, 34)**
		**Female**	**20 (17, 23)**	**25 (21, 28)**
Jan 2021 to Dec 2021	60-<70 years	Male	8 (5, 10)	22 (18, 26)
		Female	10 (7, 12)	21 (17, 25)
	70-<80 years	Male	31 (22, 40)	33 (23, 42)
		Female	24 (17, 30)	26 (19, 33)
	≥80 years	Male	43 (27, 57)	59 (41, 76)
		Female	24 (15, 32)	39 (30, 48)
	**≥60 years**	**Male**	**20 (16, 24)**	**31 (26, 35)**
		**Female**	**17 (14, 20)**	**26 (23, 30)**
Jan 2022 to Dec 2022	60-<70 years	Male	13 (9, 18)	22 (18, 26)
		Female	17 (12, 21)	21 (17, 26)
	70-<80 years	Male	59 (41, 74)	35 (24, 44)
		Female	45 (32, 56)	27 (20, 34)
	≥80 years	Male	95 (60, 127)	81 (56, 104)
		Female	54 (35, 72)	55 (41, 68)
	**≥60 years**	**Male**	**39 (32, 47)**	**34 (29, 39)**
		**Female**	**32 (27, 38)**	**29 (25, 33)**
Jan 2023 to Dec 2023	60-<70 years	Male	43 (27, 57)	78 (63, 92)
		Female	53 (39, 68)	75 (60, 89)
	70-<80 years	Male	141 (100, 179)	132 (93, 168)
		Female	107 (78, 134)	105 (77, 130)
	≥80 years	Male	286 (181, 382)	304 (210, 390)
		Female	162 (105, 216)	207 (155, 254)
	**≥60 years**	**Male**	**109 (89, 129)**	**127 (108, 146)**
		**Female**	**90 (75, 106)**	**107 (94, 122)**

RSV = respiratory syncytial virus. IFV = influenza virus. ARI = acute respiratory infections.

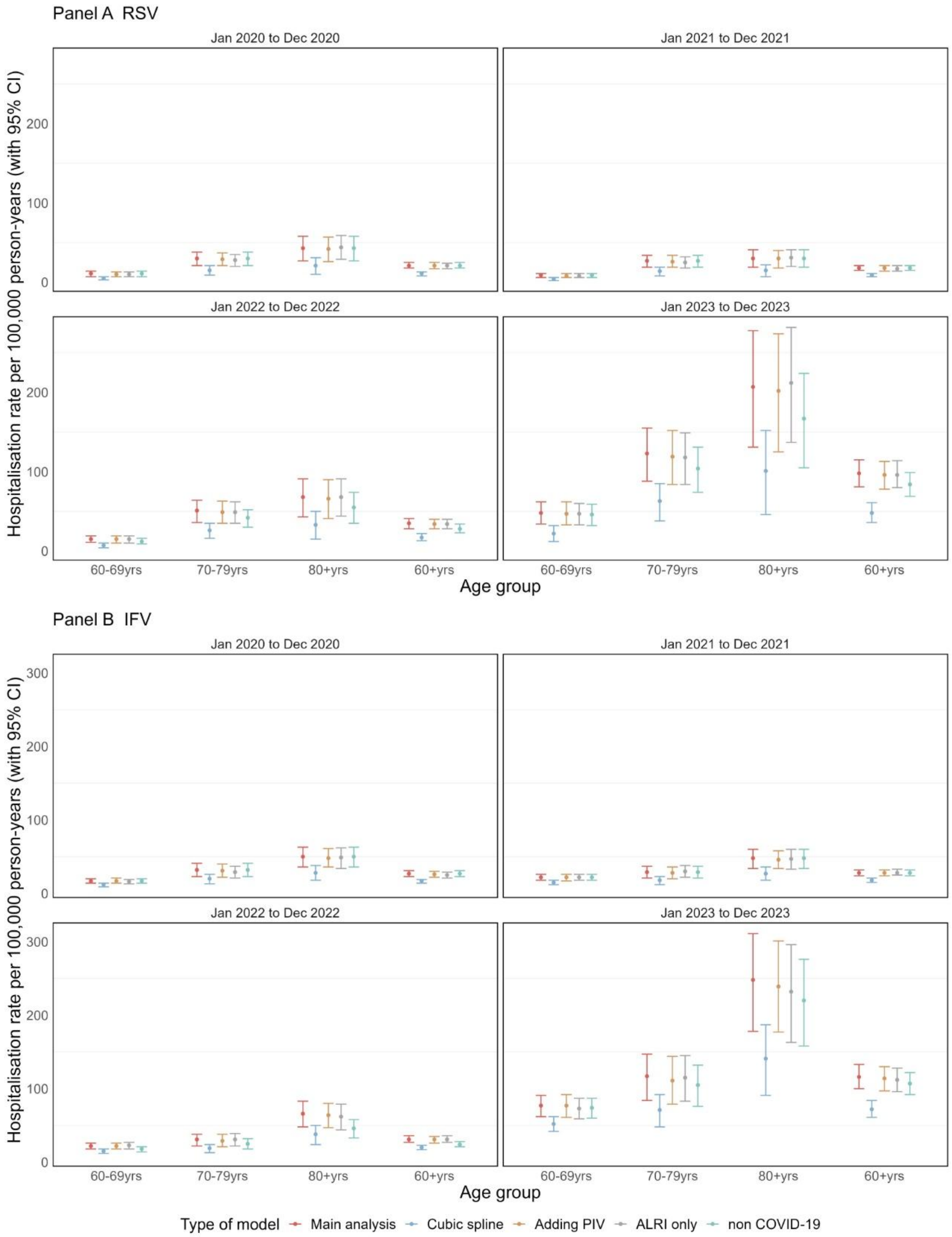

Figure S1. Comparison of estimated hospitalisation rates between main and sensitivity analyses.

RSV = respiratory syncytial virus. IFV = influenza virus. PIV = parainfluenza virus. ARI = acute respiratory infections. ALRI = acute lower respiratory infections. COVID = coronavirus disease. yrs = years.
